# Metastatic uterine histiocytic sarcoma: A rare presentation as solitary paraspinal mass

**DOI:** 10.1016/j.radcr.2025.04.100

**Published:** 2025-05-21

**Authors:** Aruba Nawaz Khattak, Aymen Shahab, Fatima Israr, Hira Yaqub, Zulfiqar Sheikh, Kashif Siddique

**Affiliations:** a Department of Radiology, Shaukat Khanum Memorial Cancer Hospital and Research Center, Lahore, Pakistan; b Department of Pathology, Shaukat Khanum Memorial Cancer Hospital and Research Center, Lahore, Pakistan

**Keywords:** Histiocytic sarcoma, Endometrial histiocystic sarcoma, Uterus, Cervix, Pelvic sidewall lymph nodes, Metastasis, Paraspinal masses, CT scan, MRI, Immunohistochemical stains

## Abstract

Histiocytic uterine sarcoma is an extremely rare tumor with aggressive clinical course characterized by the proliferation of histiocytic cells. It is a challenging entity in terms of both diagnosis and therapy. On imaging, it has nonspecific features and a broad range of differentials, depending on regional involvement. Definitive diagnosis often requires a combined approach using microscopic and pathologic analysis in conjunction with an immunohistochemical stains. We present the case of a 65 year old getting diagnosed as a case of endometrial histiocytic sarcoma developing progressive disease over the course of 1 year despite having undergone surgical resection followed by radiation therapy. The pattern of metastatic involvement down the course of her disase is unique in its presentation as a solitary paraspinal mass with no imaging evidence of visceral metastasis. Though uterine sarcomas can metastasize to unusual sites, paraspinal metastasis is almost unprecedented, emphasizing the need for thorough imaging and surveillance. With this report, we not only aim to highlight a rare metastatic presentation of uterine histiocytic sarcoma, but also to give an insight into the clinical features, diagnostic workup, and treatment challenges.

## Introduction

Histiocytic sarcoma is an extremely rare tumor posing challenges in both diagnosis and treatment with often poor prognosis [1]. Categorically, it is a hematopoietic neoplasm of unknown etiology with tumor cells demonstrating features of histiocytic differentiation both in histology and immunophenotyping. The causative factor for pathogenesis is postulated to be inactivation of p16, a regulatory tumor suppressor gene [2]. Diagnosis primarily relies on morphological and pathological characteristics; key role being played by immunohistochemistry (IHC). For extra nodal occurrence, imaging and radiologic features are particularly valuable [3]. Nonetheless, radiologic findings have rarely been reported. Management of histiocytic sarcoma is a combination of surgical resection, chemotherapy and radiotherapy. The disease itself is very aggressive with mean survival of only several months in disseminated cases. However, in localized cases, patients may survive years after primary diagnosis, if aggressively managed [4]. In terms of presentation and severity, histiocytic sarcoma displays a wide spectrum. It ranges from localized disease to multisystem involvement. Various anatomical sites can be involved including the skin, soft tissue, gastrointestinal tract, lungs, bladder, kidneys, bones, brain, and lymph nodes [5]. We present a case of histiocytic sarcoma arising from endometrium, with development of solitary para spinal metastasis. The occurrence of both is a rare entity.

## Case presentation

**Fig. 1:**
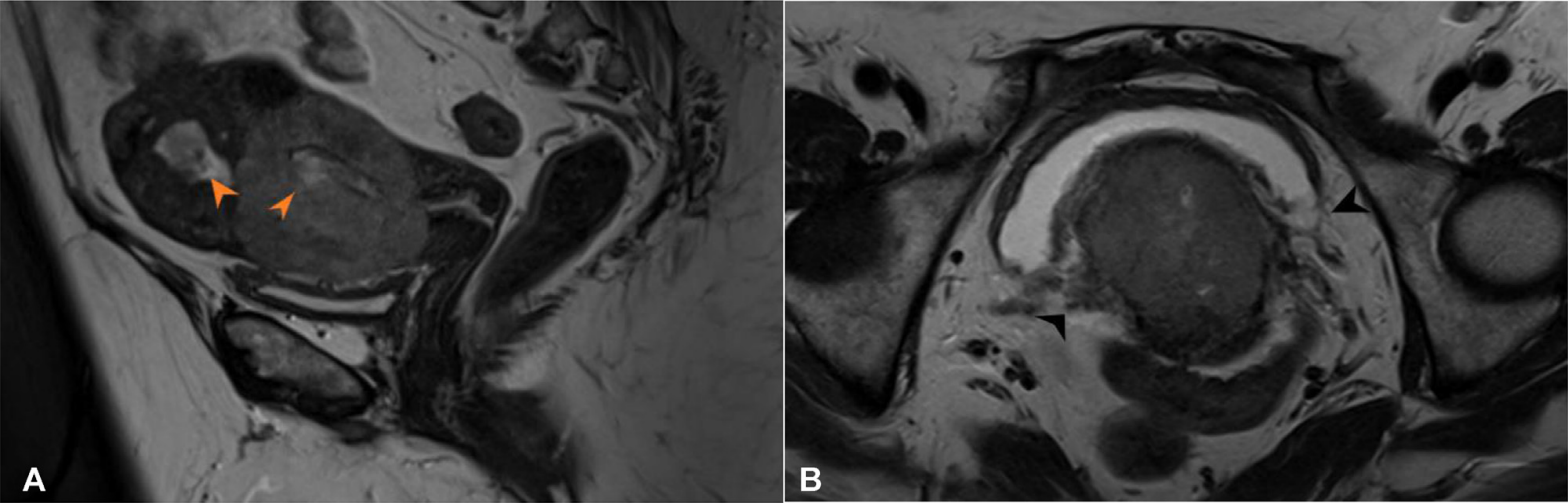
Sagittal T2 weighted (A) and Axial T2 weighted (B) images show lobulated soft tissue mass with intermediate signal intensity involving the lower uterine segment and cervix. Abnormal T2 signal is seen extending into the endometrial cavity (orange arrow heads). Early parametrial extension seen as surrounding fat stranding (black arrowheads).

**Fig. 2:**
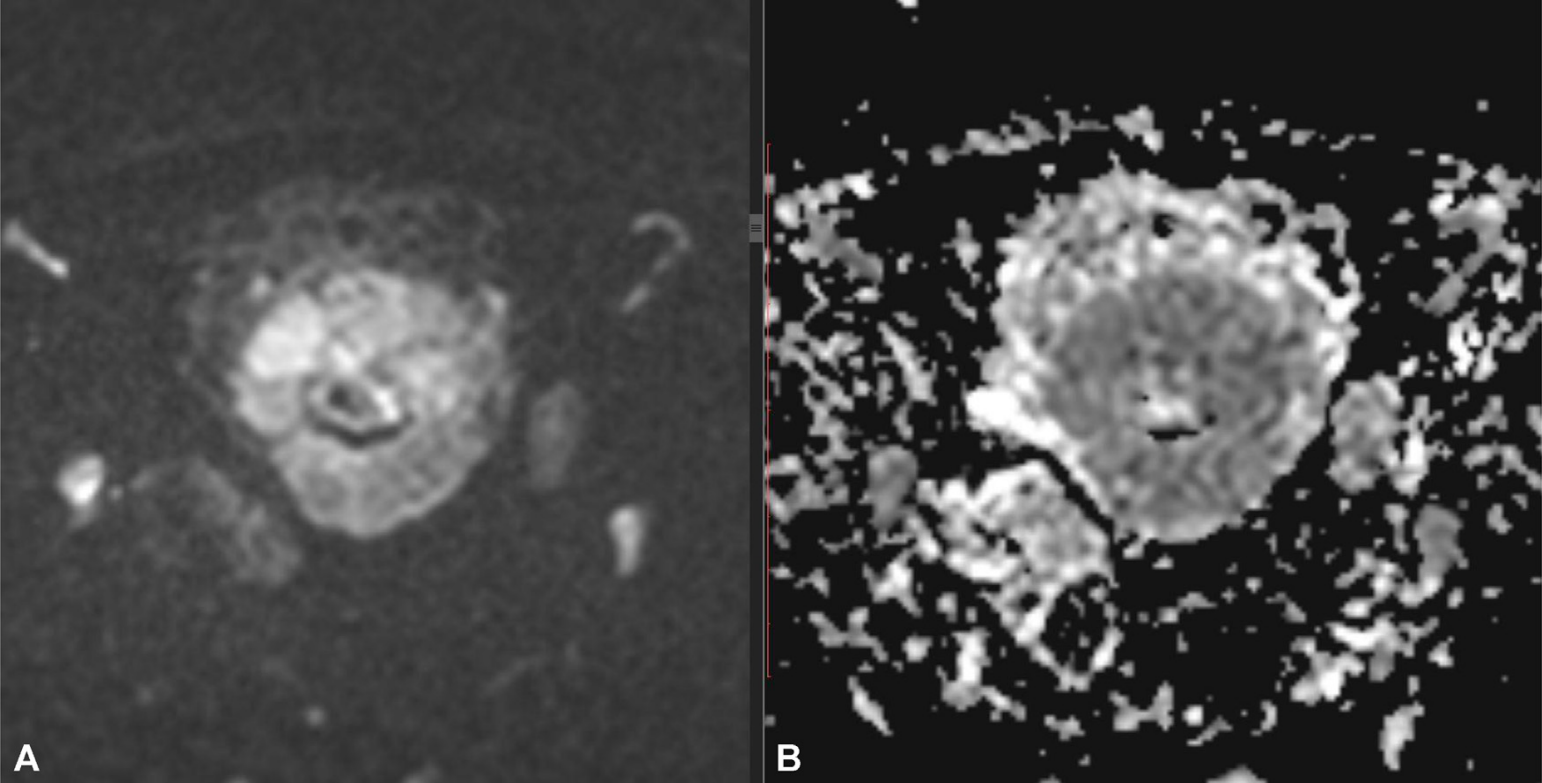
Diffusion weighted images. Infiltrative uterine mass show restricted diffusion seen as hyper intense signals on DWI and hypo intensity on ADC. Associated involved pelvic sidewall lymph nodes also show restricted diffusion.

**Fig. 3:**
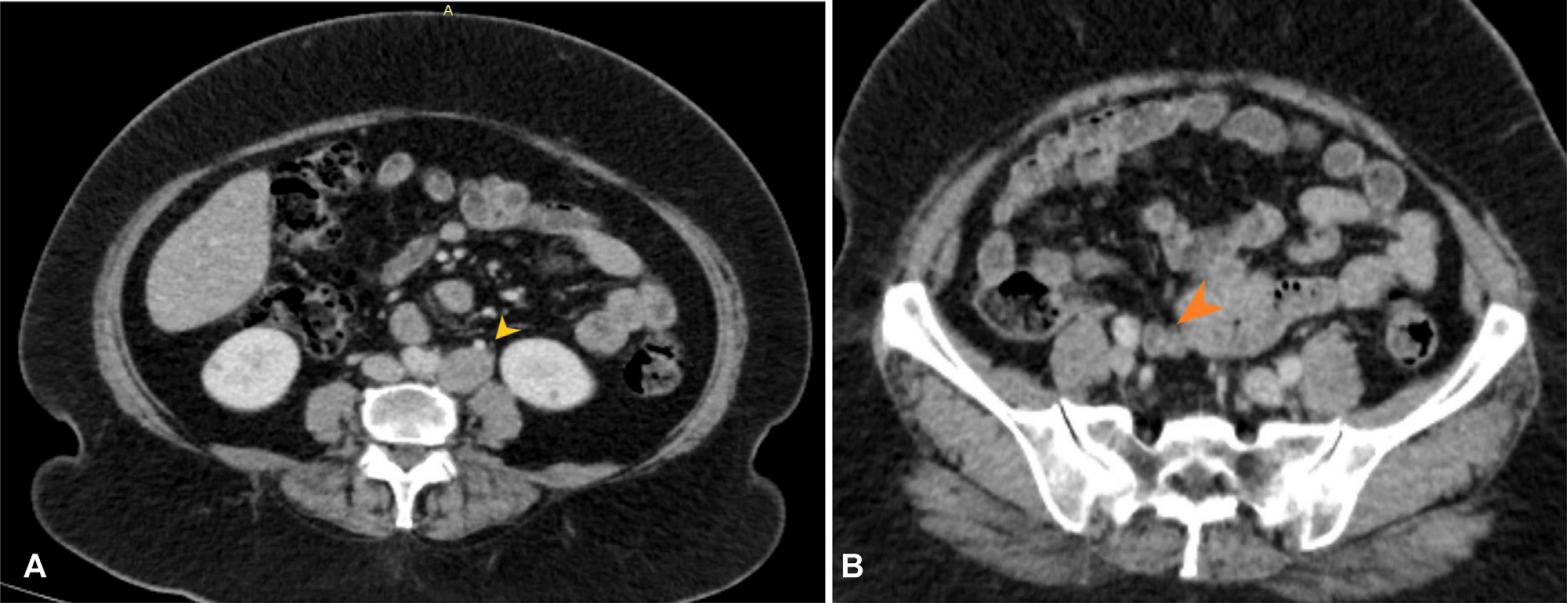
CT abdomen (soft tissue window) in axial plane show enlarged left para aortic (A, yellow arrowhead) and right common iliac lymph nodes (B, orange arrow head).

Our case is of a 65 year old woman who presented with history of postmenopausal bleeding for 1 month in March 2023. Endometrial biopsy outside hospital showed spindle cell neoplasm. Histopathology was reviewed at our hospital and patient was diagnosed to have undifferentiated malignant neoplasm favoring undifferentiated sarcoma. Patient underwent in-house MRI pelvis which showed lobulated soft tissue mass involving lower uterine segment and cervix with extension into endometrial cavity and likely early right parametrial involvement (Fig. 1, Fig. 2). Associated bilateral enlarged pelvic side wall lymph nodes. Baseline staging CT scan revealed enlarged left para aortic and right common iliac lymph nodes without any definite visceral metastasis (Fig. 3).

**Fig. 4:**
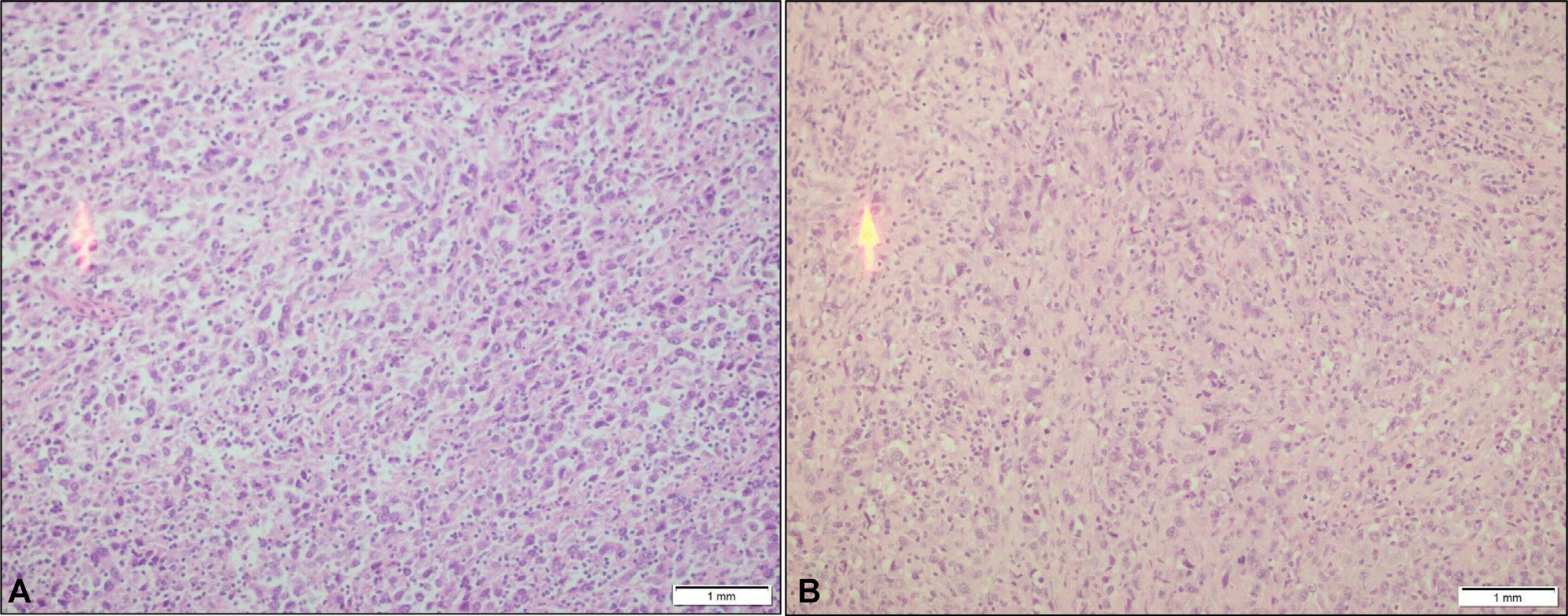
(A) High power view of endomyometrium infiltrated by histiocytic sarcoma (40x) (B)Tumor cells infiltrating cervical stroma (40x).

**Fig. 5:**
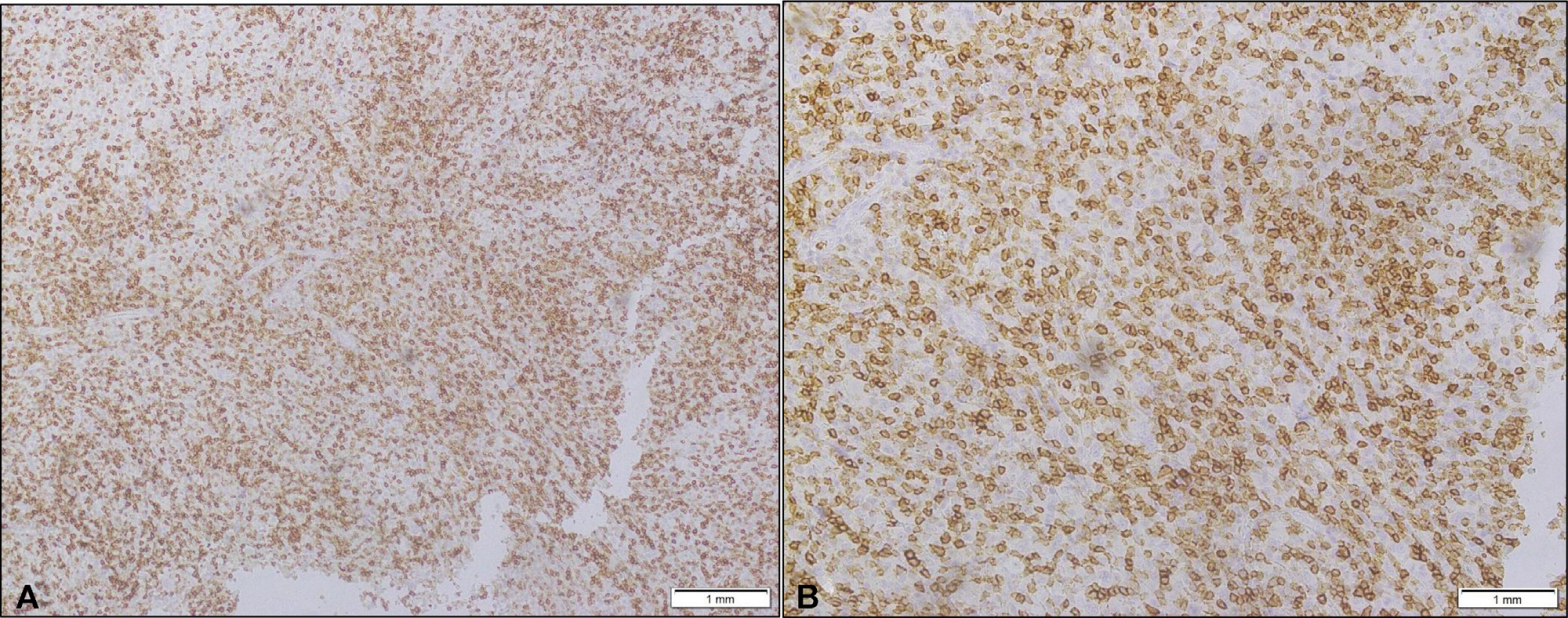
(A) CD68 showing positive membranous staining (20x) (B) CD4 showing strong and uniform membranous staining (40x).

After detailed discussion of her history, histopathology and scans, in gynecological multidisciplinary team meeting (MDT), she was booked for staging laparoscopy and surgery if resectable, with para aortic lymph node dissection. Patient underwent cystoscopy and bilateral JJ stenting with total laparoscopic hysterectomy with bilateral salpingo ophorectomy and bilateral pelvic lymph node dissection with para-aortic lymph node dissection. Post operatively she was doing well and was discharged after 2 days. Histopathological examination of hysterectomy specimen revealed a sheeted malignant neoplasm, infiltrating the endomyometrium. Tumor cells showed abundant eosinophilic cytoplasm with hyperchromatic pleomorphic nuclei and prominent nucleoli. Background showed mixed inflammation, predominantly lymphocytes seen. Tumor cells were seen invading cervix as well (Fig. 4). A battery of immunohistochemical stains was applied to determine the lineage of tumor cells. Cytokeratin,p63 and CK8/18 (for epithelial origin), LCA (for lymphoid neoplasms), CD30 (for anaplastic lymphoma), CD138(for plasma cell tumors) S100 (for neural tumors), SOX 10 and HMB 45 (for melanocytic tumors), Synaptophysin and INSM1 (for neuroendocrine tumors), Desmin and Smooth muscle actin (for smooth muscle tumors), CD10, Cyclin D1 and PR (for endometrial stromal tumors) and INI /SMARCB1 (for undifferentiated carcinoma and sarcoma) were all negative . Markers of histiocytic differentiation i.e. CD 4, CD 68 and CD163 were all positive (Fig. 5). The final histopathological diagnosis was of endometrial histiocytic sarcoma—high grade pT1b.

This was followed up by her PET CT imaging which did not show any avid residual disease or distant metastasis. Later on, her case was discussed in lymphoma MDT. After review, the team members concluded her treatment in accordance with case series in UPTODATE, where 14 cases were reviewed and best outcome was with surgery followed by radiation therapy (XRT) to involved site and follow up imaging in 3-6 months. Over the course of next few months, her radiation therapy was completed with a dose of 45Gy in 25 fractions to pelvis. Follow up CT chest, abdomen and pelvis did not show any residual or recurrent disease or distant metastasis.

**Fig. 6:**
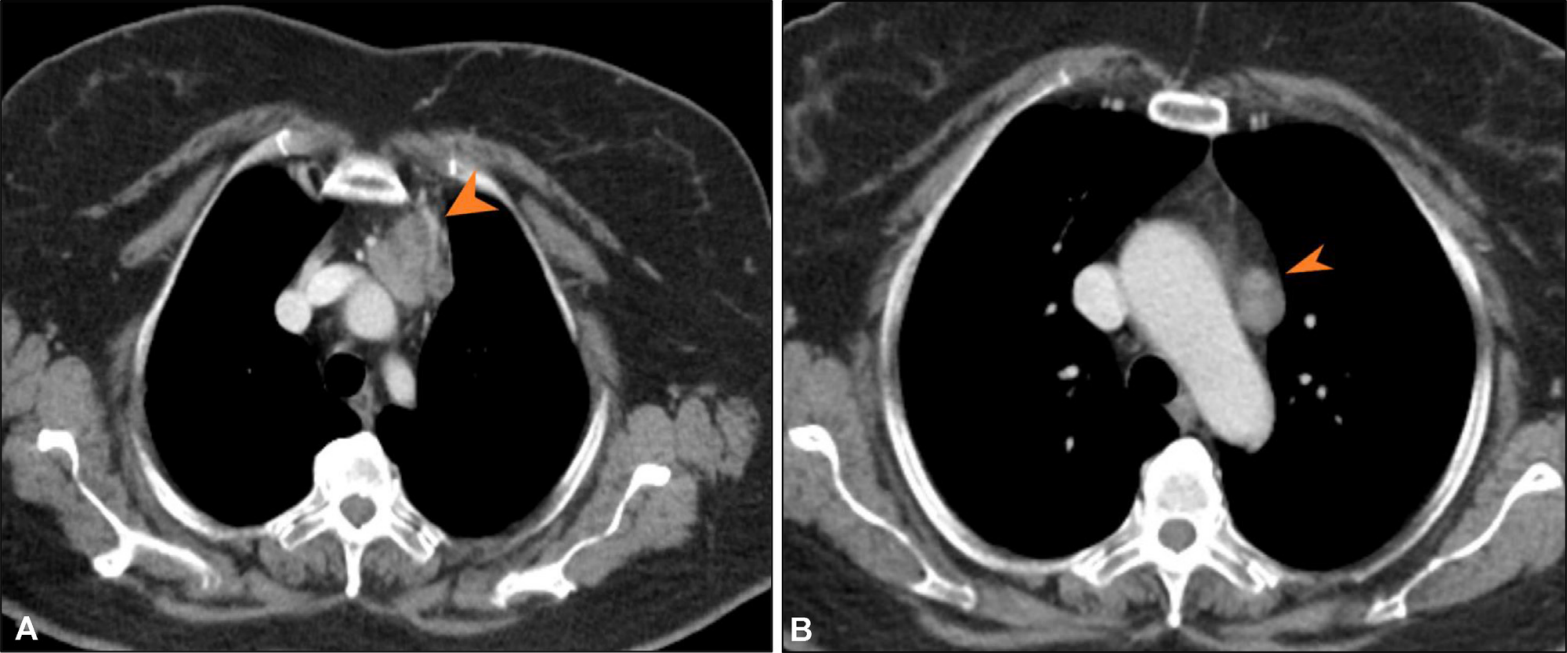
CT scan chest axial cuts (A and B) with soft tissue window show interval enlargement of mediastinal lymph nodes (arrowheads).

**Fig. 7:**
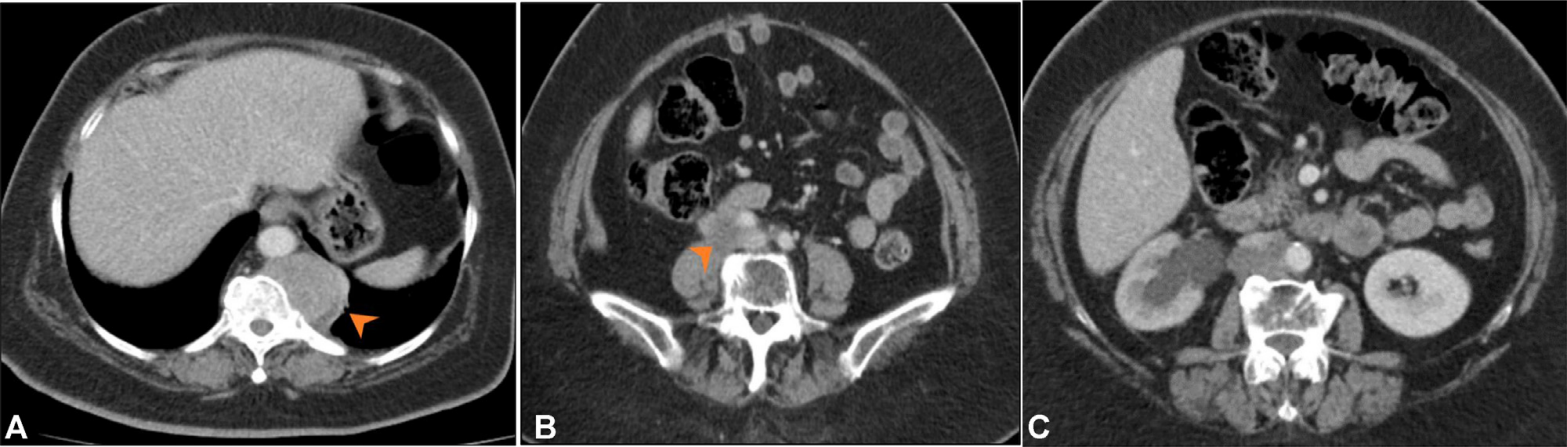
CT scan abdomen axial cuts with soft tissue window reveal left para spinal mass lesion (A). Right retroperitoneal lymph nodes at infra renal level (B) with mass effect on right mid ureter and resultant upstream right hydroureteronephrosis (C).

The patient was on surveillance until September 2024, when she presented to Emergency Assessment Room (EAR) with complaints of severe backache. Her CT scan revealed interval development of right sided moderate hydronephrosis and paraspinal soft tissue mass at the level of T9 to T12 extending into neural foramina (Fig. 7). Above diaphragm, there was interval enlargement of mediastinal lymph nodes, however, no visceral metastasis were seen and there was no local recurrence (Fig. 6).

She was planned for right percutaneous nephrostomy, however pre procedural imaging revealed minimal hydronephrosis not amenable to the procedure. She was started on oral analgesia and her case was discussed in gynecological MDT again. The recommendation of the meeting were to proceed with MRI Spine, attempt interventional radiology guided biopsy of paravertebral mass and book with radiation oncologist.

**Fig. 8:**
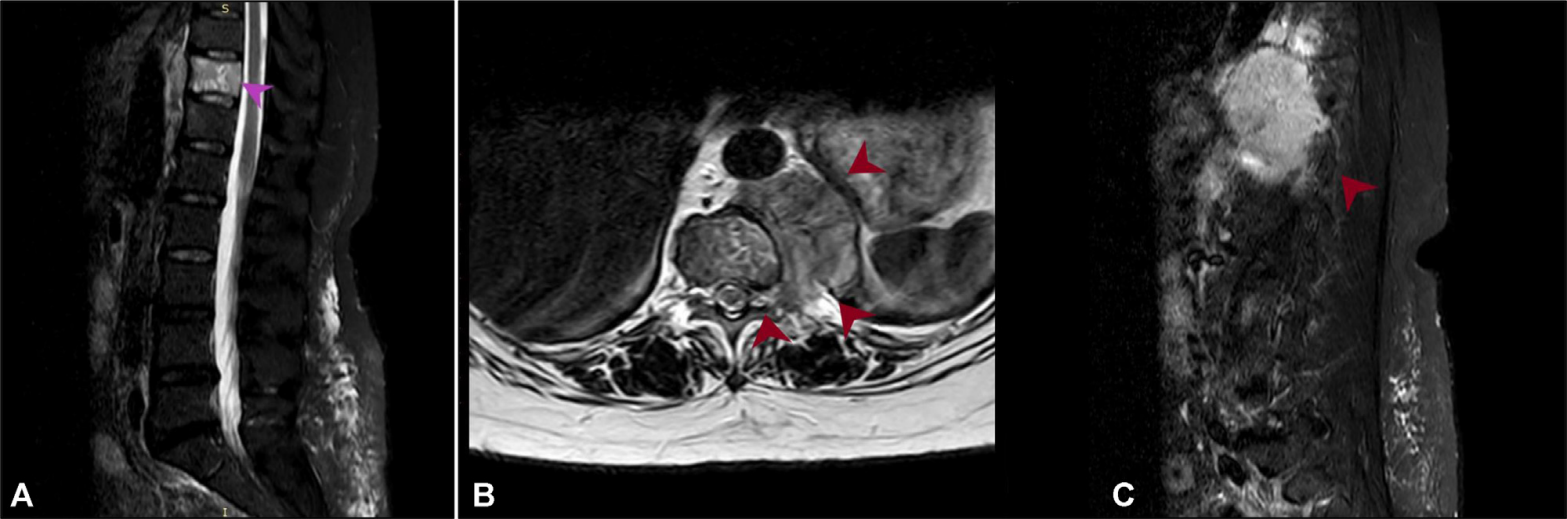
(A) Sagittal STIR image shows metastatic deposit at T10 vertebral body (pink arrowhead). This patient had sacralization of L5 vertebral body. (B) Axial T2 weighted and (C) sagittal STIR images, large left paravertebral soft tissue mass extending from T9 to T12 vertebral body. Anteriorly the mass is encroaching onto fat planes with abdominal aorta at this level. Posteriorly, there is extension into the left T10/T11 intervertebral foramen (red arrowheads).

MRI spine showed osseous metastasis, in particular involving T10 vertebral body with associated large left paravertebral soft tissue mass not causing cord compression (Fig. 8). After undergoing checkup from neurooncologist, she was planned for CT guided biopsy of paravertebral mass followed by MDT discussion.

Patient underwent CT guided core biopsy which turned out to be a metastatic deposit compatible with known history of histiocytic sarcoma. This was discussed at length in neurology and sarcoma MDT and unanimous consensus of palliative management was coined. She was booked for palliative radiotherapy of 30Gy in 10 fractions to the T10 paravertebral soft tissue mass and paraaortic mass. Follow up PET CT imaging revealed increase in size of right paraaortic mass and interval development of hyper metabolic, left supraclavicular and mediastinal lymph nodes with focal marrow uptake in T11 vertebral body culminating into progressive disease.

## Discussion

Histiocytic sarcoma (HS) is well known to be a rare and aggressive malignancy originating from histiocytic cells [6]. It represents less than 1% of all hematolymphoid neoplasms [2]. There are fewer than 40 cases reported in the literature [7]. It is a nonlangerhans histiocytic disorder characterized by high-grade, pleomorphic histiocytic cells and can arise in any tissue or organ. The disease poses a significant diagnostic challenge not only due to its rarity, but also because it often mimics a wide variety of other neoplasms, like lymphomas, sarcomas, and carcinomas [1]. Histopathological review with immunohistochemical markers such as CD4, CD163, CD68, and lysozyme help in distinguishing HS from its mimics [8].

For better treatment purposes, initial evaluation focuses on histologic diagnosis, extent and site of disease and performance status of the patient. Keeping in view, guidelines in UpToDate Version 28.0, staging strategies include laboratory workup (CBC, electrolytes, liver and renal function tests). Positron emission tomography (PET)/computed tomography (CT) to document organ involvement. HS appears to be PET-avid in small case series [9]. Additionally, it includes bone marrow biopsies for patients with cytopenias or hemophagocytosis.

Most of the case reports published on various presentations of histiocytic sarcoma have shown disease occurrence in 5th and 6th decades [3].

Our patient is also a 65 year old lady who presented with postmenopausal bleeding. This is a common but very nonspecific symptom that can indicate various gynecological pathologies, including endometrial carcinoma, fibroids, and, less frequently, uterine sarcomas. In this patient, an initial biopsy outside the hospital suggested a spindle cell neoplasm, which was later diagnosed as an undifferentiated malignant neoplasm favoring undifferentiated sarcoma at our Hospital. This diagnosis paved way for further imaging which eventually confirmed the disease process centered as a lobulated mass involving the lower uterine segment, cervix, and associated enlarged pelvic sidewall lymph nodes.

The diagnostic challenge in this patient was the rarity of endometrial histiocytic sarcoma. The fact that this neoplasm shows overlapping features with other sarcomas and malignancies such as lymphoma, melanoma and poorly differentiated carcinomas, made it essential to use a combination of imaging and immunohistochemical markers to arrive at a correct diagnosis. In our case, imaging played an essential role in assessing the disease's extent and staging. The clinical presentation of this patient was in line with what is commonly observed in early-stage HS, with a pelvic mass, bilateral pelvic sidewall lymph nodes, and no evidence of visceral metastasis. A comprehensive multidisciplinary team (MDT) discussion involving gynecologists, oncologists, radiologists, and pathologists was pivotal for diagnostic confirmation and for planning the course of further management**.**

Endometrial sarcoma, particularly HS, is considered an aggressive disease with a poor prognosis, often requiring multimodal treatment. While surgery is typically the first line of treatment, the lack of randomized clinical trials for this rare condition makes it difficult to establish a standardized treatment regimen. Total hysterectomy, bilateral salpingo-oophorectomy, and lymph node dissection are commonly performed and recommended for localized disease. Role of adjuvant chemotherapy and radiation remains controversial. For disseminated disease, lymphoma-type regimens (cyclophosphamide, doxorubicin, vincristine, and prednisone (CHOP), ifosfamide and etoposide±carboplatin (ICE), adriamycin, bleomycin, vinblastine and dacarbazine (ABVD)) are often employed. However, no prospective data on outcomes are available [10]. Our patient underwent a successful surgical resection, with histopathology confirming high-grade histiocytic sarcoma (pT1b). Postoperative imaging with the PET scan revealing no residual disease or distant metastasis was also satisfactory as this is considered to be a positive sign for patients who undergo complete surgical resection.

In the limited available literature, it has been established that there is no standardized treatment for HS, and especially in disseminated disease, the clinical course is overly aggressive with a dismal outcome [11]. Unfortunately, despite the initial success in management, the patient’s clinical course took a significant turn when she presented with back pain and signs of obstructive uropathy. On subsequent imaging, there was metastatic involvement of the spine (T10 vertebral body), retroperitoneal lymphadenopathy and hydronephrosis. This recurrence underlined the aggressive nature of histiocytic sarcoma, which frequently leads to distant metastases even after initial complete resection. The metastatic spread to the vertebrae and mediastinal lymph nodes in this patient is in keeping with the highly malignant nature of the disease, as described in similar cases by Fachetti et al. [12]. The management of metastatic HS remains challenging, and while surgery can provide palliation for certain metastatic sites, the role of radiation therapy (XRT) is also crucial in addressing local disease recurrence.

In our case, following an MDT discussion it was decided to pursue palliative radiotherapy for the T10 paravertebral mass and paraaortic lymph nodes. Palliative radiation is usually employed for symptomatic relief and to control the local spread of the disease, particularly in patients with nonresectable or metastatic disease [2]. After radiation therapy, follow-up imaging revealed progression of the disease, including increased size of the right paraaortic mass and new hypermetabolic left supraclavicular and mediastinal lymph nodes, further confirming disease progression.

There is no established management guideline for advanced or metastatic disease and treatment often follows the principles established for other aggressive sarcomas or lymphomas. Systemic therapies such as chemotherapy or immunotherapy have shown promise in some cases, but further research is needed to identify effective treatment regimens. Despite having undergone radiotherapy, our patient had disease progression which highlights the need for coming up with new management strategies.

Furthermore, this case emphasizes the need for and importance of comprehensive surveillance following treatment for Histocytic Sarcoma. For early detection of metastasis and recurrence, patients should undergo regular clinical and imaging based follow-ups. This can help in timely intervention and hence improve outcomes. Being rare, each case adds valuable insight into the clinical presentation, treatment options, and prognosis of this challenging malignancy.

## Conclusion

This case of histiocytic sarcoma highlights the challenges in diagnosing and managing rare, aggressive malignancies. The multidisciplinary approach to diagnosis and treatment, including surgical resection followed by radiation therapy, was crucial in the patient’s initial management. However, the subsequent metastatic recurrence and the shift toward palliative care emphasize the need for ongoing research to improve treatment outcomes and develop standardized care pathways for patients with advanced histiocytic sarcoma.

## Author contributions

A.N.K (Aruba Nawaz Khattak) conceived and designed the case report, F.I (Fatima Israr) handled documental workup related to patient consent and IRB approval, H.Y (Hira Yaqub) and Z.S (Zulfiqar Sheikh) provided pathology input. A.A (Aymen Shahab) helped in data collection. A.N.K (Aruba Nawaz Khattak) performed the literature review and wrote the manuscript. K.S (Kashif Siddique), A.A and F.I reviewed and critically revised the manuscript. All authors have approved the final manuscript.

## Patient consent

This case report was conducted with the approval of the institutional review board (EX-04-12-24-02) and written informed consent for the publication of this case report was obtained from the patient’s son. All patient data was de-identified to maintain confidentiality.
